# An ITPA Enzyme with Improved Substrate Selectivity

**DOI:** 10.1007/s10930-023-10162-0

**Published:** 2023-12-08

**Authors:** Nicholas E. Burgis, Kandise VanWormer, Devin Robbins, Jonathan Smith

**Affiliations:** 1 Department of Chemistry, Biochemistry & Physics, Eastern Washington University, Cheney, WA 99004, USA

**Keywords:** ITPA, ITP, Thiopurine, Ribavirin, Inosine, DEE 35

## Abstract

Recent clinical data have identified infant patients with lethal ITPA deficiencies. ITPA is known to modulate ITP concentrations in cells and has a critical function in neural development which is not understood. Polymorphism of the *ITPA* gene affects outcomes for both ribavirin and thiopurine based therapies and nearly one third of the human population is thought to harbor *ITPA* polymorphism. In a previous site-directed mutagenesis alanine screen of the ITPA substrate selectivity pocket, we identified the ITPA mutant, E22A, as a gain-of function mutant with enhanced ITP hydrolysis activity. Here we report a rational enzyme engineering experiment to investigate the biochemical properties of position 22 ITPA mutants and find that the E22D ITPA has two- and four-fold improved substrate selectivity for ITP over the canonical purine triphosphates ATP and GTP, respectively, while maintaining biological activity. The novel E22D ITPA should be considered as a platform for further development of ITPA therapies.

## 1 Introduction

The ITPA protein is essential for life in humans due to its central role in removing inosine triphosphate (ITP) from cellular nucleotide pools [1, 2]. It is expressed in all major organs in adult humans with the highest expression observed in the heart, liver, thyroid and thymus [3]. Data indicate that ITPA supports a critical role in proper neuron function, and that this activity is required for sustained viability of an individual [2, 4–7]. ITPA preferentially hydrolyzes ITP to inosine monophosphate and has equivalent activity with the deoxyribonucleotide and ribonucleotide forms [8]. ITPA also hydrolyzes the canonical nucleotides (deoxy)adenosine triphosphate ((d)ATP) and (deoxy)guanosine triphosphate ((d)GTP), but at a 50- and 30-fold reduced rate, respectively [9].

Over 300 distinct polymorphisms have been identified in the human *ITPA* gene [2, 10–16], which encodes the ITPA protein, and it has been estimated that nearly a third of the global population may harbor *ITPA* polymorphism that is associated with decreased ITPA activity [17–19]. One of the more common polymorphisms, c.94 C > A (p.Pro32Thr), affects about 5% of humans [20] and is associated with poor outcomes for patients undergoing thiopurine treatment [21], but improved outcomes for patients undergoing hepatitis C treatment with ribavirin [22–24].

To date, 45 patients have been reported to have very rare mutations which causes severe ITPA deficiency and high mortality in the first few years of life [2, 4–7, 25–29]. Several of the first patients identified were diagnosed with an early infantile condition termed developmental and epileptic encephalopathy 35 (DEE 35; MIM# 616,647) [2, 27], while two later patients were diagnosed with a Martsolf-like syndrome with lethal infantile dilated cardiomyopathy [5]. Other studies have identified the *ITPA* gene as a recessive infantile epilepsy causative or related gene [6, 26, 28]. The molecular mechanism of pathogenesis in patients with severe ITPA deficiency is unknown. Deoxyinosine is not detected in DNA from cells of patients who have homozygous null mutations for *ITPA* [5], presumably due to DNA mismatch repair activity [30], suggesting the mechanism of pathogenesis is not at the DNA level. Instead, it has been postulated that consequential effects on RNA function and/or metabolism [31] or interference of ITP with other nucleotide functions, such as interfering with ATP or GTP-binding proteins may occur [2, 32, 33].

We are not currently aware of any treatments that directly address ITPA defects. Instead a personalized medicine approach has been adopted to modulate drug dosage in response to known *ITPA* polymorphism, such as in the case of thiopurine [34] or ribavirin treatment [35]. Treatment for DEE 35 aims to control the epileptic seizures through diet and medication, however patients have poor outcomes and are often refractory to anti-seizure medicine [6].

As drug delivery and gene replacement technologies improve [36–40], it is pertinent to consider the development of therapies that directly address ITPA deficiencies. From a protein engineering approach, the aim for ITPA is to engineer an enzyme with equal or better stability and more efficient activity with ITP, but less efficient activity with ATP/GTP [41]. This can be accomplished by developing an enzyme with equal or reduced binding of ITP, while maintaining equivalent or increased ITP catalysis, but also causing binding of ATP/GTP to stay the same or weaken, while ultimately reducing catalysis for ATP/ GTP [41].

For this study, we used the rational design method [42] to interrogate the substrate binding pocket of ITPA to improve biological activity for the protein. Previously, we published results of an alanine screen we performed to better understand the role of critical amino acid residues in the substrate binding pocket of ITPA [43]. Many of our results followed predictable outcomes based on existing structural information [44]. However, we also identified mutants with improved activity with ITP. From this study, the E22A ITPA emerged as a good lead for rational design in our protein engineering efforts, however, protein stability appeared to be compromised [43]. Here we report our investigation of additional Glu 22 ITPA mutants. Of the E22 mutants tested, our data indicate the E22D ITPA mutant has the most robust enhancement in substrate specificity for ITP compared to the canonical nucleotide triphosphates and performs the best in two complementation tests.

## 2 Materials & Methods

### 2.1 Plasmid Construction

pET28a- and pBJH-based mutant plasmids were constructed as described previously [43] using pET28a-*ITPA* plasmid as a template [9].

### 2.2 Purification

Wild-type and mutant proteins were overexpressed with a hexahistidine tag as described previously [43]. Frozen pET28a transformed *Escherichia coli* cell pellets were resuspended in 20 mM phosphate, 0.5 M NaCl 10 mM imidazole buffer at pH 7.4 (Buffer A) and sonicated as before [43]. A BioLogic LP System (www.bio-rad.com) was used to load the cleared lysate onto a 1 mL Ni^2+^ charged HisTrap HP affinity chromatography column. After washing with 10–15 column volumes of Buffer A, recombinant protein was eluted with Buffer A containing 500 mM imidazole. Fractions were analyzed by SDS-PAGE [45] and those containing purified target protein were selected for dialysis against 20 mM Tris–HCl pH 7.4, 100 mM NaCl, 10 mM MgCl_2_ and 1 mM 1,4-dithiothreitol (Buffer B) at 4 °C. The final protein preparations were stored in 50% glycerol at −20 °C (supplemental Figure S1). Protein concentration was quantified using a NanoDrop 2000 (www.thermofisher.com). Typical yields were 1–2 mg for mutant proteins and 6 mg for wild-type.

### 2.3 Specific Activity

Specific activity was measured similar to [46]. Briefly, reaction volumes were 300 μl and contained 20 mM Tris-HCl at pH 7.4, 100 mM NaCl, 10 mM MgCl_2_ and 0.2 pmol enzyme with 100 μM ITP or 100 pmol enzyme with 200 μM GTP. Reactions were pre-incubated at 37°C and, after addition of enzyme, reactions proceeded for 10 and 20 min for ITP and GTP, respectively. After incubation at 37°C, reactions were stopped by addition of an equal volume of 2% sodium dodecyl sulfate solution. Reaction tubes were mixed and centrifuged and an aliquot of the supernatant was removed for analysis. Next, reaction products were separated on a Nucleogen 60 – 7 DEAE column (www.mn-net.com) using a ThermoFisher UltiMate 3000 HPLC system at a flow rate of 0.6 ml/ min and buffer containing 75 mM sodium phosphate, pH 6.4, 5% acetonitirile and 0.4 mM EDTA. Product formation was measured by absorbance at 248 and 253 nm for ITP and GTP reactions, respectively, using an UltiMate 3000 VWD-3400RS UV detector. Reactions were run in duplicate with an average velocity calculated and three independent replicates performed. Statistically significant differences were determined using Student’s *t*-test [47].

### 2.4 Enzyme Kinetics

Michaelis-Menten parameters were determined with an assay similar to the specific activity assay above. Wildtype and the three mutant enzymes were assayed side by side in 100 μl volumes using eight different substrate concentrations each. Substrate concentration ranged from 5 to 100 μM ITP, 50–750 μM GTP, and 200–600 μM ATP. Each reaction was pre-incubated at 37 °C for 10 min, with reaction times of 10 min for ITP, 60 min for GTP and 90 min for ATP. Each reaction contained 0.2 pmol enzyme for ITP, 20 pmol enzyme for GTP and 100 pmol enzyme for ATP with product formation of ATP being monitored at 259 nm. At least three independent experiments were performed for each enzyme/substrate pair. The Enzyme Kinetics module of Sigma Plot software was used to determine kinetic parameters and perform best fit analysis. Kinetic parameters are reported as average values ± standard error.

### 2.5 HAP Cytotoxicity Assays

N-6-hydroxylaminopurine (HAP) cytotoxicity assays were performed as described in [43] using 0, 10 and 25 μg/ml HAP.

### 2.6 *recA* Complementation Assays

Synthetic lethal complementation tests were performed as described in [43], here measurements were taken every two hours.

## 3 Results

### 3.1 Specific Activity

**Fig. 1:**
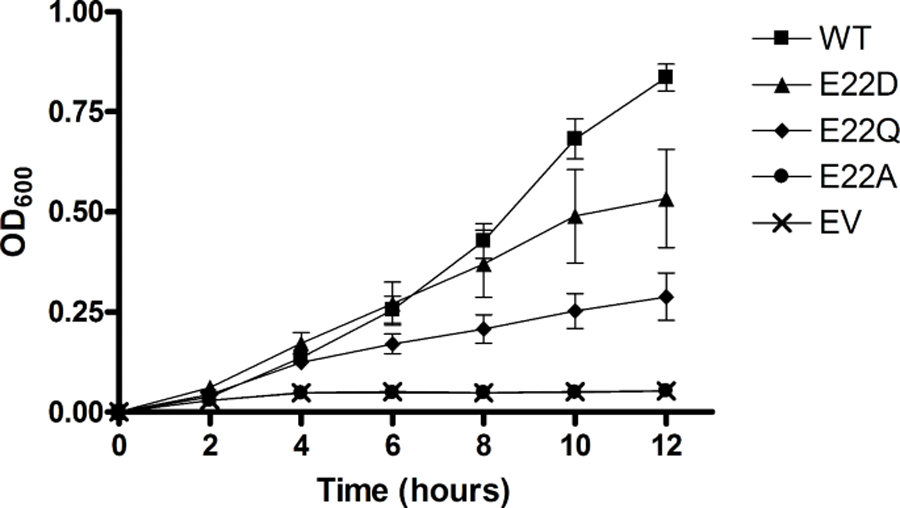
Specific activity measurements for wild-type (WT) and E22 ITPA mutants with ITP (**A**) and GTP (**B**). Asterisks indicate statistically significant difference from wild-type activity (P < 0.05)

Figure 1A shows specific activity measurements for wildtype and E22 mutant ITPA with ITP as a substrate. As originally observed, E22A showed significantly higher activity, improving activity over wild type by about 50%, but E22D had activity similar to wild-type and the E22Q activity was significantly lower. The activities for all three mutants with GTP as substrate were at least three times less than wild-type (Fig. 1B). Notably, the wildtype activity with ITP is roughly 1000 times greater than it is with GTP.

### 3.2 Enzyme Kinetics

Enzyme kinetics experiments were performed as described above with purified recombinant wild-type ITPA and the E22A, E22D and E22Q mutant ITPA using ITP, GTP, and ATP as substrates (Table 1). Results with wild-type were consistent with previously published results given different techniques and assay conditions [9]. With ITP as a substrate, the *K*_*M*_ values for the mutant enzymes ranged from equivalent to wild-type, to about three-fold higher. Notably, the E22D mutant had a *K*_*M*_ value equivalent to wild-type, indicating this change does not affect ITPA binding, while the E22A and E22Q mutants had increased *K*_*M*_ values, indicates reduced ITP binding. These mutations may cause a distortion in the pocket that hinders optimal hydrogen bond formation within the specificity pocket and/or between enzyme and substrate [44]. Remarkably, the average rate of catalysis increased for all three mutants. The increase in *k*_*cat*_ ranged from about a 1/3 increase to a nearly two-fold increase over wild-type. However, the specificity constants (*k*_*cat*_/*K*_*M*_) were fairly equivalent to wild-type for all mutants.

With GTP as a substrate, the *K*_*M*_ for E22A is much lower than wild-type and the other mutants, with *K*_*M*_ values for wild-type, E22D, and E22Q all between about 500–700 μM. For E22A, the two-fold reduction in average *K*_*M*_ suggests that the larger substrate selectivity pocket accommodates the bulkier guanine base better than the other enzymes. Both *k*_*cat*_ and *k*_*cat*_/*K*_*M*_ are considerably reduced for all mutants with GTP as a substrate. In fact, all three mutants display a reduction in catalysis that is at least five-fold lower than wild-type.

The data with ATP as a substrate has more error due to it being such a poor substrate. With ATP, average *K*_*M*_ values are similar to wild-type for E22A and E22Q, but over three-fold higher for the E22D mutant. *k*_*cat*_ values are quite low for all enzymes with the E22A and E22Q mutants having an average value lower than wild-type, but the average for E22D is elevated two-fold. Compared to wild-type, the *k*_*cat*_/*K*_*M*_ value for the E22Q mutant is equivalent, but it is about two-fold less for the E22A and E22D mutants. To our knowledge this is the first report of enzyme kinetics data with ATP as a substrate for human ITPA.

### 3.3 Substrate Specificity Enhancement

With ITPA having such a critical role in removing ITP from cellular nucleotide pools [48], it is important to consider how ITPA handles ITP compared to the canonical nucleotides. To approximate enhancement of selectivity for ITP over the canonical nucleotides, the level of enhancement for ITP substrate specificity (ratio of wild-type *k*_*cat*_/*K*_*M*_ to mutant *k*_*cat*_/*K*_*M*_) and the level of decrease for GTP/ATP substrate specificity was compared for each mutant. Table 2 shows that the substrate enhancement for ITP compared to GTP is enhanced roughly five-fold for the mutants. ATP enhancement was less pronounced, and the maximal effect was two-fold for the E22D mutant. While the E22D mutant has the lowest enhancement with respect to GTP, the enhancement with respect to ATP is the highest of the three mutants. Altogether, for the E22D mutant, this indicates that the substrate preference for ITP compared to GTP and ATP increased over four-fold and two-fold, respectively.

### 3.4 Hill Equation with ATP

A best fit analysis indicated our data fit the Hill equation more favorably than the Michaelis-Menten equation when ATP was the substrate. Velocity vs. [S] plots for each enzyme with ATP as a substrate are shown in Figure S2 with the trend lines fit to the Hill equation. Here we observed positive cooperativity (*n* > 1) for all enzymes [49] and a substantial reduction in ATP hydrolysis for the E22A and E22D mutants, but not for the more conservative E22Q mutant, consistent with our results using the standard Michaelis-Menten equation (Table 1). For wild-type, we observed a Hill coefficient (*n*) of 1.8, and kinetic parameters in range of those observed using the Michaelis-Menten equation (Table 3). For the Hill analysis, the *K*_*D*_ values for all enzymes are much lower than the *K*_*M*_ values calculated with the Michaelis-Menten equation, but the specificity constants are similar to those observed using the Michaelis-Menten equation. Interestingly, the Hill coefficients for all three mutants are above two. ITPA has been shown to be a dimer in solution [3], but is also known to interact with itself [50], so the higher *n* values may be a result of complex formation. It should be noted that this is the first report of cooperativity for human ITPA, and a significant cooperative effect is only observed with ATP as a substrate ( n ≈ 1 for ITP and GTP, data not shown), however cooperativity was reported for the *E. coli* ortholog with multiple substrates [51].

### 3.5 Biological Assays – Complementation Tests

#### 3.5.1 HAP Sensitivity

**Fig. 2:**
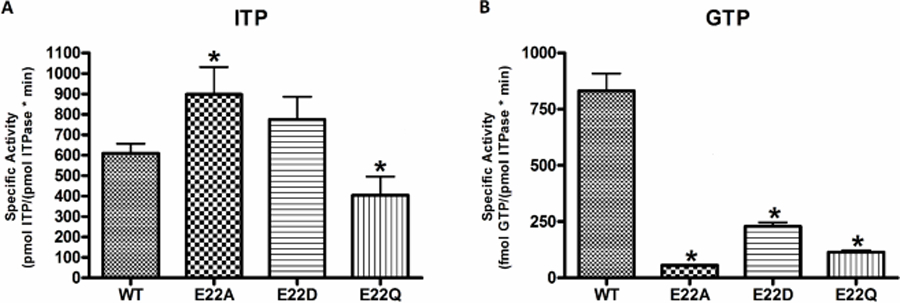
HAP sensitivity complementation test. *E. coli* cells were transformed with empty-vector (EV) or plasmid containing WT or mutant *ITPA* and appropriate dilutions were plated on media containing indicated concentrations of HAP. Cells were incubated at 37 °C for 48 h

The fitness of each mutant was tested by constitutively expressing each mutant protein via a plasmid that has been transformed into *E. coli* cells that are engineered to be sensitive to the base analog HAP (Fig. 2). At the higher HAP concentration, the E22D mutant has a survival rate comparable to wild-type, while the E22Q mutant has an intermediate survival rate and the E22A mutant has a survival rate equivalent to empty vector. This result shows the E22D mutant offers a high level of protection against the toxic effects of HAP and behaves like wild-type in vivo.

#### 3.5.2 Temperature Sensitivity

**Fig. 3:**
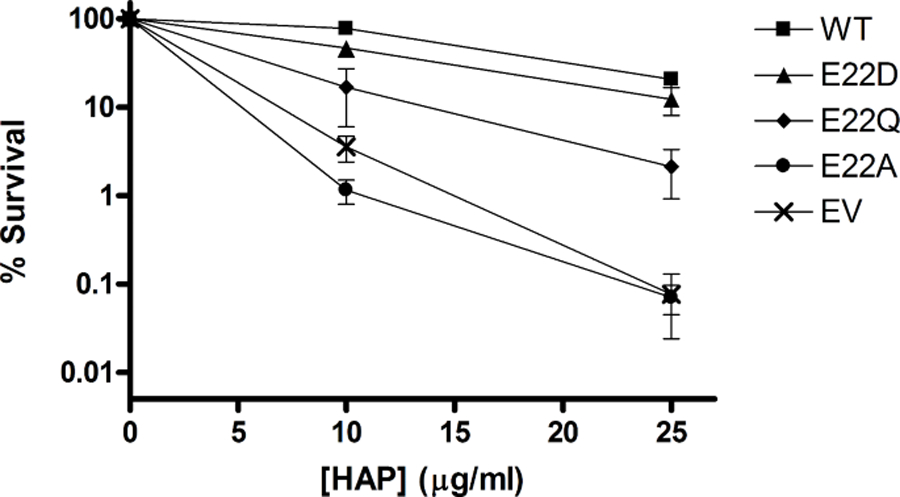
Temperature sensitivity complementation test. *E. coli* cells were transformed with EV or plasmid containing WT or mutant *ITPA* at 30 °C. Fresh overnight cultures were then diluted into broth pre-warmed at 42 °C and cell density was monitored at OD_600_.

The fitness of each mutant was also tested by constitutively expressing each mutant protein via a plasmid that has been transformed into *E. coli* cells that are engineered to be sensitive to endogenous ITP when cells are grown at an elevated temperature (Fig. 3). After 12 h growth at 42 °C, the wild-type culture was in the late log-phase of growth and had reached an optical density significantly higher than any of the mutants. The E22D mutant again outperformed the E22Q mutant while the E22A mutant was similar to empty vector. The E22A results for both complementation tests are consistent with previously published data. Serial dilution plating assays produced similar results (data not shown). These data demonstrate that the E22D mutant can provide some protection from endogenous ITP, even at elevated temperatures.

## 4 Discussion

Genuine ITPA deficiency has been described for multiple patients and generally results in death at a young age [2, 4–7, 25–29]. Currently no treatment is available for this condition indicating a need to address this orphan disease. To date this is the only report we know of which provides a general platform to develop methodologies to reverse clinically observed ITPA deficiency. We postulate that delivery of enhanced ITPA to affected patients via CRISPR or mRNA therapies may be valuable for producing a therapeutic effect and suggest that the E22D ITPA serve as a platform to consider when developing ITPA therapies.

Data suggest that the main role of ITPA is to reduce the accumulation of ITP in cells [1, 32, 48, 52] and that loss of ITPA activity can result in imbalances in nucleotide pools which are detrimental to proper cell function, and impacts neural function significantly [2, 5]. Patients who are c.94 C>A (p.Pro32Thr) homozygous have no detectable ITPase activity in red blood cells (RBCs) and the level of ITP generally rises to well over 100 μM [14]. Compared to a calculated value of 3 μM for RBCs in general [53, 54], this suggests that ITPA significantly reduces the level of ITP in cells, and that in the absence of ITPA activity, ITP concentrations will rise to levels similar to canonical NTPs. It is hypothesized that high levels of ITP may interfere with cellular mechanisms that require ATP and GTP, such as G-proteins or actomyosin, and RNA metabolism/function [2, 31–33].

Jimmerson et al. have determined the concentrations of ATP, GTP and ITP in peripheral blood mononuclear cells (PBMC) to be 1,303, 264 and 1.5 pmol/10^6^ cells, respectively [53]. Given the volume of PBMCs to be about 195 fl. [55, 56], the intracellular concentrations of ATP, GTP and ITP are calculated to be about 6.9, 1.4 and 0.0077 mM, respectively, where the ratio of ATP: ITP is about 870 and the ratio of GTP:ITP is about 180. For the canonical NTPs, these calculations are within established ranges. For instance, literature searches of ATP concentration in a wide range of biological samples determined that millimolar concentrations of ATP are widespread throughout biology, with an average value of 4.41 mM and a range of at least 2 to 7 mM ATP [57]. Looking at predominantly mammalian cells and fluids, literature values are 3,152 +/− 1,698 μM for ATP and 468 +/− 224 μM for GTP [58]. Because the ratio of ITP concentration to canonical NTP concentration is two- to three-orders of magnitude, improving the selectivity of ITP over ATP/GTP is critical to boosting E22D ITP hydrolysis activity and is expected to give E22D an advantage when handling ITP over ATP and GTP in intracellular conditions.

Further consideration of the kinetic constants for these mutants gives additional insight into ITPA mechanics (Table 1). Under our experimental conditions (low rate of catalysis), *K*_*M*_ measurements are reflective of substrate binding [41, 49] and *K*_*M*_ values for the wild-type have a trend consistent with ATP, GTP and ITP concentrations in the cell [53]. Because Glu-22 is located in the substrate selectivity pocket [44], the mutants studied are not expected to alter the enzyme’s catalytic mechanism, so changes in *k*_*cat*_ are likely driven by altered binding rather than a defect in catalysis. Notably, with ITP as a substrate, the specificity constants are similar for all four enzymes, but the binding and catalytic rate parameters are not. The discrepancies between the parameters for E22A/E22Q mutants and wild-type highlight the tradeoff between binding ability and catalytic optimization and support the idea that the ability to bind ITP at low micromolar concentrations is evolutionarily favored over catalytic efficiency [49].

While the enhancement of enzyme activity with ITP is minimal, the major improvement for the E22D mutant is that it has less non-productive activity with the canonical nucleotides ATP and GTP. With GTP, the E22D and wildtype *K*_*M*_ values are roughly equivalent, hence substrate binding would be roughly equivalent, but the mutant has five-fold lower rate of GTP hydrolysis (Table 1). Therefore, GTP would bind in the mutant’s active site at a rate equivalent to wild-type, however this binding would be five-fold less productive than wild-type. While this would render the enzyme unavailable to act on ITP while GTP is bound unproductively, the fact that the *K*_*M*_ values are equivalent between E22D and wild-type, suggests that an equivalent number of ITPA active sites would have GTP bound for both enzymes, hence, E22D is expected to outperform wildtype for ITP hydrolysis if relative ITP and GTP concentrations are the same [41, 49].

For the E22D mutant, the amino acid at position 22 extends 1 carbon bond length shorter (than wild-type) into the substrate selectivity pocket [44]. Therefore, it can likely accommodate the larger GTP base better than wild-type, but this results in a five-fold reduced rate of catalysis. With a somewhat larger binding pocket, there could be additional conformational flexibility for bound GTP or perturbations of GTP binding at the specificity pocket could result in poor alignment of the scissile phosphodiester bond in the enzyme’s active site. Either scenario would result in reduced enzyme efficiency.

For ATP, the *K*_*M*_ value for E22D is 3.7-fold higher than wild-type, indicating that binding affinity for the enzyme is greatly reduced (Table 1). This shifts the *K*_*M*_ value to that of intracellular ATP concentrations [57], while the *k*_*cat*_ values indicate the mutant hydrolyzes ATP at a rate about two-fold less than wild-type. Given that enzymes are thought to be at half their maximal activity when substrate concentration is equal to *K*_*M*_ [49], it can be deduced that ATP would occupy the active-site of E22D ITPA about 50% less than it would the wild-type ITPA active site at intracellular ATP concentrations [53, 57, 58]. Therefore, minimized binding of ATP is the major factor driving the substrate specificity enhancement with respect to ATP for E22D. Overall, ATP is expected to bind the active site less often and be hydrolyzed at a rate less than wild-type. This would allow the E22D mutant to be more available to bind ITP, because the active site would not be occupied by ATP as often. Because the ratio of ITP concentration to canonical NTP concentration is two- to three-orders of magnitude [53], improving the selectivity of ITP over ATP/GTP is critical to boosting E22D activity and is expected to give E22D an advantage when handling ITP over ATP and GTP in intracellular conditions. Altogether, the data suggest that for E22D, both GTP and ATP would bind to the enzyme with equal or lesser affinity and catalysis would be less efficient, thus reducing the amount of nonspecific activity with the canonical nucleotides.

The cooperativity observed with ATP as a substrate is notable. Because ITPA has been shown to interact with itself [50], it is possible the ITPA dimers could complex together under certain circumstances. Although we did not observe cooperativity for ITP or GTP, it cannot be ruled out. For ATP, the *k*_*cat*_ and specificity constant values are generally an order of magnitude less than GTP, indicating that ITPA activity with ATP is extremely low. Therefore, a high concentration of ATP was needed to stay well above detection limits of the assay. As a result, it is possible that at such high concentration of substrate, and low rate of catalysis/specific activity, there is considerable nonproductive substrate binding which could result in both monomer active sites occupied and facilitating cross-talk between monomers which produces a cooperative effect with ATP. If that is the case, it is likely that ITP and GTP produce a cooperative effect as well, but that it is not great enough to observe under our experimental conditions.

A major challenge of protein engineering is developing a protein that is sufficiently stable in the environment/application where it is needed [41, 59]. While the enzyme kinetics data alone did not clearly discern one mutant that outperforms the others, the complementation data showed the E22D mutant has the best protective activity in vivo, and therefore is likely the most stable mutant studied. The complementation data indicate that E22D is capable of wild-type like activity in cells under normal 37 °C growth conditions (see Fig. 2). While indirect, this is good evidence that E22D has protein stability equivalent to the wild-type enzyme over the length of the 48-hour assay. However, for the second complementation test, where temperatures are elevated to 42 °C for 12 h, the E22D mutant does retain much of the protective activity in cells, but at a level that is reduced compared to wild-type (Fig. 3). Overall, this suggests that the E22D mutant has stability that is equivalent to wild-type under normal cellular conditions.

*ITPA* polymorphism affects a substantial number of individuals [1] and can affect clinical outcomes [21, 24] or result in severe developmental delays and infantile death [2, 4–7, 25–28]. To date, no therapies have been developed to address the clinical complications or treat this orphan disease. The idea that about 30% of the world population may have reduced ITPA activity [17–19], makes it plausible that a subset of the population may benefit from ITPA therapy under certain circumstances, such as during thiopurine treatment [21]. Here we report the first effort to develop an ITPA enzyme with enhanced activity and present data demonstrating the E22D ITPA has a two- to four-fold substrate specificity enhancement and functions equivalent to wild-type under normal cellular conditions.

Our rational design experiments have yielded the E22D ITPA as a starting point for further experimentation aimed at alleviating ITPA defects. For instance, the E22D mutant could serve as a platform for directed evolution experiments, such as phage display or computational methods [60]. Additionally, an enhanced protein could be beneficial as gene delivery/replacement therapies are developed as an enhanced protein may be necessary to deliver the effective dosage or overcome challenges with delivery or expression should they be encountered. Future work is needed to better quantify stability of the E22D mutant as well as direct NTP competition tests to better quantify the enhancement of E22D for ITP hydrolysis over ATP/GTP hydrolysis.

## Supplementary Material

supplementary material

## Figures and Tables

**Table 1:** Kinetic constants for E22 ITPA mutants

Enzyme	Substrate	*K_M_(μM)*	*k_cat_(s^−1^)*	*k_cat_/K_M_(mM^−1^s^−1^)*
wild-type	ITP	20.8 ± 4.3	16.6 ± 1.2	798 ± 176
E22A	ITP	60.9 ± 13	32.1 ± 3.6	526 ± 127
E22D	ITP	24.6 ± 4.8	21.5 ± 1.6	872 ± 183
E22Q	ITP	41 ± 2.4	32.1 ± 0.6	782 ± 48
wild-type	GTP	665 ± 81	0.036 ± 0.0026	0.0542 ± 0.0077
E22A	GTP	292 ± 36	0.00204 ± 0.0001	0.007 ± 0.0010
E22D	GTP	529 ± 45	0.0071 ± 0.0003	0.0134 ± 0.0013
E22Q	GTP	719 ± 65	0.00672 ± 0.0004	0.00935 ± 0.0010
wild-type	ATP	1487 ± 544	0.0022 ± 0.0006	0.00147 ± 0.0007
E22A	ATP	1016 ± 805	0.000667 ± 0.0004	0.000656 ± 0.0006
E22D	ATP	5511 ± 10,012	0.00447 ± 0.0075	0.00081 ± 0.0020
E22Q	ATP	792 ± 322	0.00135 ± 0.0004	0.0017 ± 0.0008

**Table 2:** Substrate specificity enhancement

Enzyme	ITP enhancement	GTP decrease	ATP decrease	Substrate specificity	Substrate specificity

				enhancement wrt GTP	enhancement wrt ATP
E22A	0.659	7.743	2.143	5.1-fold	1.4-fold
E22D	1.093	4.045	1.875	4.4-fold	2.0-fold
E22Q	0.980	5.766	0.882	5.7-fold	0.86-fold

**Table 3:** Kinetic constants with Hill analysis

Enzyme	Substrate	*n*	*K_D_(μM)*	*k_cat_(s^−1^)*	*k_cat_/K_M_(mM^−1^s^−1^)*	ATP decrease
wild-type	ATP	1.8	336.8	0.00083	0.0025	
E22A	ATP	3.5	244.2	0.00025	0.001	2.5
E22D	ATP	2.5	318.3	0.0005	0.0016	1.6
E22Q	ATP	2.1	259	0.00065	0.0025	0

## Abbreviations

ITP: Inosine triphosphate
(d)ATP: (Deoxy)adenosine triphosphate
(d)GTP: (Deoxy)guanosine triphosphate
DEE 35: Epileptic encephalopathy 35
RBCs: Red blood cells
PBMCs: Peripheral blood mononuclear cells
