# Supplementary material for: An ITPA Enzyme with Improved Substrate Selectivity
Source: Protein J. Author manuscript; Available in PMC 2024 Mar 8. (PMC10901923; doi:10.1007/s10930-023-10162-0)

An ITPA enzyme with improved substrate selectivity. *The Protein Journal.*

Nicholas E. Burgis, Kandise VanWormer, Devin Robbins, Jonathan Smith.

Eastern Washington University, Cheney, WA, USA. Corresponding author: nburgis@ewu.edu

Figure S1. SDS-PAGE of purified wild-type and mutant recombinant ITPA. SDS-PAGE was performed as previously described (43). 4 μg total protein was loaded for each prep. Lane 1, Precision Plus Protein Standard (Bio-Rad, sizes to left in kDa); lane 2, WT; lane 3, E22A; lane 4, E22D; lane 5, E22Q.

Fig S1.

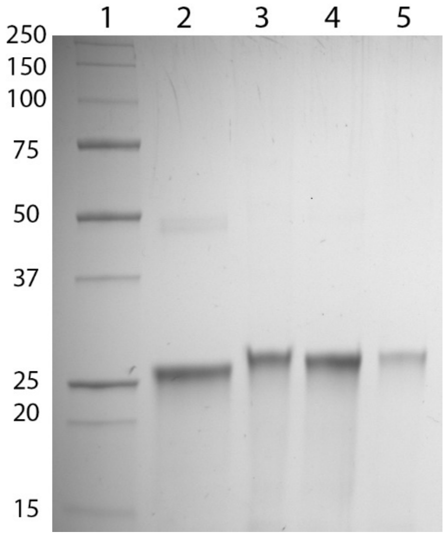

An ITPA enzyme with improved substrate selectivity. *The Protein Journal.*

Nicholas E. Burgis, Kandise VanWormer, Devin Robbins, Jonathan Smith.

Eastern Washington University, Cheney, WA, USA. Corresponding author: nburgis@ewu.edu

Figure S2. Velocity vs. [S] plots for wild-type and mutant ITPA with ATP as a substrate. Trend lines fit to the Hill Equation with R^2 values noted. A, WT; B, E22A; C, E22D, D, E22Q.

Fig S2.

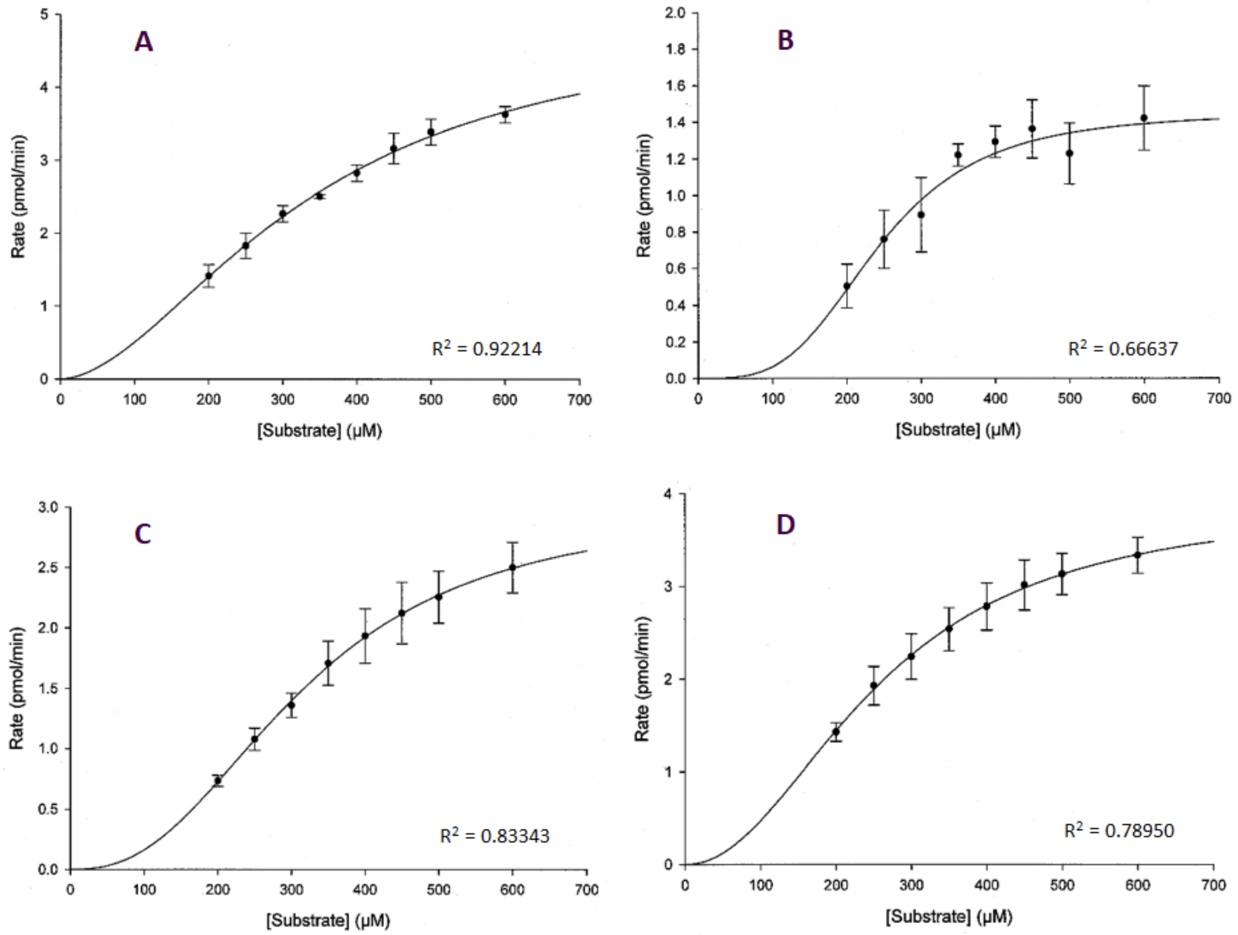

Supplement: supplementary material [file NIHMS1968433-supplement-supplementary_material.pdf]
